# Diagnostic accuracy of interferon-gamma release assays for diagnosis of smear-negative pulmonary tuberculosis: a systematic review and meta-analysis

**DOI:** 10.1186/s12890-022-02013-y

**Published:** 2022-06-06

**Authors:** Tananchai Petnak, Dararat Eksombatchai, Supavit Chesdachai, Ploypin Lertjitbanjong, Pahnwat Taweesedt, Angsupat Pornchai, Charat Thongprayoon, Larry J. Prokop, Zhen Wang

**Affiliations:** 1 Division of Pulmonary and Pulmonary Critical Care Medicine, Department of Medicine, Faculty of Medicine Ramathibodi Hospital, Mahidol University, 270 Ramathibodi Hospital, Rama VI Road, Ratchathewi, Bangkok, 10400 Thailand; 2 Division of Infectious Diseases, Department of Medicine, Mayo Clinic, Rochester, MN USA; 3 Division of Pulmonary, Critical Care, and Sleep Medicine, Department of Medicine, University of Tennessee Health Science Center, Memphis, TN USA; 4 Department of Pulmonary Medicine, Corpus Christi Medical Center, Corpus Christi, TX USA; 5 Division of Pulmonary and Critical Care Medicine, Department of Medicine, Mayo Clinic, Rochester, MN USA; 6 Division of Nephrology and Hypertension, Department of Medicine, Mayo Clinic, Rochester, MN USA; 7 Mayo Clinic Libraries, Mayo Clinic, Rochester, MN USA; 8 The Mayo Clinic Robert D. and Patricia E. Kern Center for the Science of Health Care Delivery, Mayo Clinic, Rochester, MN USA; 9 Evidence-Based Practice Research Program, Mayo Clinic, Rochester, MN USA

**Keywords:** Diagnosis, Interferon-gamma release assays, Meta-analysis, Sensitivity, Specificity, Smear-negative, Tuberculosis

## Abstract

**Introduction:**

The diagnosis of smear-negative pulmonary tuberculosis (SNPTB) is challenging. Interferon gamma-release assays (IGRAs) may be helpful in early diagnosis among these patients resulting in prompt treatment and favorable outcomes.

**Methods:**

We performed a comprehensive search from each databases’ inception to April 5, 2021. The studies that provided sufficient data regarding the sensitivity and specificity of IGRAs included QuantiFERON-TB Gold In-Tube (QFT-GIT), T-SPOT.TB, or QuantiFERON-TB Gold Plus for diagnosis of SNPTB were included.

**Results:**

Of 1,312 studies screened, 16 studies were included; 11 QFT-GIT, 2 T-SPOT.TB, and 3 QFT-GIT and T-SPOT.TB. For diagnosis of SNPTB, QFT-GIT had sensitivity of 0.77 (95% CI 0.71–0.82), specificity of 0.70 (95% CI 0.58–0.80), diagnostic odds ratio (DOR) of 8.03 (95% CI 4.51–14.31), positive likelihood ratio (LR) of 2.61 (95% CI 1.80–3.80), negative LR of 0.33 (95% CI 0.25–0.42), and area under receiver operating characteristic (AUROC) of 0.81 (95% CI 0.77–0.84). T-SPOT.TB had sensitivity of 0.74 (95% CI 0.71–0.78), specificity of 0.71 (95% CI 0.49–0.86), DOR of 6.96 (95% CI 2.31–20.98), positive LR of 2.53 (95% CI 1.26–5.07), negative LR of 0.36 (95% CI 0.24–0.55), and AUROC of 0.77 (95% CI 0.73–0.80). The specificity seemed lower in the subgroup analyses of studies from high tuberculosis burden counties compared to the studies from low tuberculosis burden.

**Conclusion:**

IGRAs do have insufficient diagnostic performance for SNPTB. However, the tests are still helpful to exclude tuberculosis among patients with low pre-test probability.

*Registry*: PROSPERO: CRD42021274653.

**Supplementary Information:**

The online version contains supplementary material available at 10.1186/s12890-022-02013-y.

## Introduction

Tuberculosis is a major health problem and one of the leading causes of death worldwide. Estimated deaths related to tuberculosis were 1.3 million in 2020 [1]. Among patients with pulmonary tuberculosis, 20–50% of patients had negative sputum acid-fast stain—known as smear-negative pulmonary tuberculosis (SNPTB) [2–10]. Although the gold standard for pulmonary tuberculosis diagnosis is the isolation of *Mycobacterium tuberculosis* from the culture, sputum collection may not be feasible or adequate in some situations, particularly in SNPTB patients. Thus, the diagnosis of SNPTB remains a challenging clinical conundrum. Furthermore, delayed tuberculosis diagnosis leads to disease progression and increase in community spread.

Interferon-gamma release assays (IGRAs) are one of the diagnostic methods that might be helpful to diagnose SNPTB. IGRAs, including QuantiFERON-TB Gold In-Tube test (QFT-GIT), QuantiFERON-TB Gold Plus (QFT-plus), and T-SPOT.TB, are *in-vitro* blood tests detecting T-cell released interferon-gamma (IFN-γ) stimulated by *M. tuberculosis* antigen. These tests have been approved by the U.S. Food and Drug Administration (FDA) to diagnose latent tuberculosis infection (LTBI) [11]. To date, IGRAs have been increasingly used to diagnose LTBI. IGRAs appear to have a stronger predictive value than tuberculin skin tests to detect developing of later active tuberculosis [12].

However, the role of IGRAs in diagnosing active tuberculosis remains unclear. Previous systematic reviews and meta-analyses focused on the diagnostic accuracy of IGRAs for diagnosis of active pulmonary tuberculosis, regardless of sputum smear results [13–16] and extra-pulmonary tuberculosis [17, 18], demonstrated moderate diagnostic performance. The role of IGRAs on SNPTB has not been well described. Thus, we conducted a systematic review and meta-analysis to explore the diagnostic accuracy of IGRAs for the diagnosis of smear-negative pulmonary tuberculosis.

## Methods

This systematic review and meta-analysis was conducted and reported according to the Preferred Reporting Items for Systematic Reviews and Meta-analyses (PRISMA) statement. The protocol was registered with PROSPERO (CRD42021274653).

### Search strategy and eligibility criteria

We performed a comprehensive search of databases from each database’s inception to April 5, 2021, without language restriction. The databases included Ovid MEDLINE(R) and Epub Ahead of Print, In-Process & Other Non-Indexed Citations, and Daily, Ovid EMBASE, Ovid Cochrane Central Register of Controlled Trials, and Scopus. The search strategy was designed and conducted by an experienced librarian (LP) with input from the study’s principal investigator. Controlled vocabulary supplemented with keywords was used to search sensitivity/specificity of IGRAs for active tuberculosis disease in adult patients. The actual strategy listing all search terms used and how they are combined is available in Additional file 1: Table S1.

Studies were included if those: (1) studied the diagnostic accuracy of IGRA tests, including QFT-GIT, T-SPOT.TB, or QFT-plus, for diagnosis of active pulmonary tuberculosis (2) provided sufficient information to evaluate the sensitivity and specificity of IGRA tests for diagnosis of SNPTB and (3) included adult participants with age ≥ 15 years old. Studies were excluded if those: (1) did not follow the manufactory instructions or cut-off values (≥ 0.35 IU/ml for QFT-GIT and QFT-plus, and ≥ 6 spots for T-SPOT.TB) (2) performed IGRA tests from specimens other than blood (3) included patients with latent tuberculosis infection in the analyses (4) included patients with ongoing anti-tuberculosis drug for > 14 days (5) performed IGRA tests as the second sequential test relied on the result of the first test (6) included < 10 of patients with SNPTB and (7) the full articles could not be accessed.

### Data extraction

Two authors (AP and SC) independently reviewed the titles and abstracts of all articles retrieved from the systematic search to exclude irrelevant studies. In case of disagreement, the discordant articles were included in the full-text review. The full articles of included studies from the first step were independently reviewed by two authors (PL and PT) to select eligible studies and abstract data. The reviewers resolved disagreements regarding study selection and data abstraction by discussion. The reviewers also manually reviewed the references of included studies and the previous systematic reviews to identify additional eligible studies. The reliability of study selection was assessed using the percent of agreement and $\kappa$ statistic.

The following information was abstracted from each study, including author names, year of publication, study design, the country in which each study was conducted, type of IGRA test, and participant characteristics (number of participants, age, sex, proportion of confirmed pulmonary tuberculosis patients, proportion of patients with smear-negative pulmonary tuberculosis, participant immune status, history of previous tuberculosis infection, and history of BCG vaccination).

### Outcome

The primary outcomes were the diagnostic accuracy variables, including sensitivity, specificity, positive likelihood ratio (LR), negative LR, and diagnostics odds ratio (DOR). Diagnostic accuracy measures were abstracted, including true positive, true negative, false positive, and false negative for each IGRA test and reference test.

### Study quality assessment

The risk of bias in each eligible study was independently evaluated by two authors (PL and PT) using the revised Quality Assessment of Diagnostic Accuracy Studies (QUADAS-2) tool. Disagreements between two reviewers were settled by the discussion with the third reviewer (TP). We defined studies with low risk of bias if they were judged as having low risk of bias for all domains of risk of bias evaluation [19].

### Statistical analysis

We used the symmetric hierarchical summary receiver operating characteristic (HSROC) models to jointly estimate sensitivity and specificity, positive and negative likelihood ratios, and DOR [20]. We drew the HSROC curves based on the estimates and included sensitivity and specificity reported by the included studies. The area under receiver operating characteristic (AUROC) was also evaluated for each test. We were unable to pool estimates when the number of studies was less than 4. We were also unable to examine potential publication bias by evaluating funnel plots symmetry and Deeks funnel plot asymmetry tests since the number of studies was not large enough (< 20). We conducted the following pre-specified subgroup analyses: (1) studies having low risk vs. at risk of bias and (2) studies conducted in high vs. low tuberculosis burden countries. The subgroup analysis among T-SPOT.TB was unable to perform according to the low number of studies. Since patients with HIV infection and immunocompromised status were associated with false-negative IGRA tests [12, 13], we did the sensitivity analysis by excluding studies conducted on patients with HIV infection or immunocompromised status [21, 22]. Stata version 17 (StataCorp LLC, College Station, TX) was used in all statistical analyses.

## Result

### Study selection and study characteristics

**Fig. 1:**
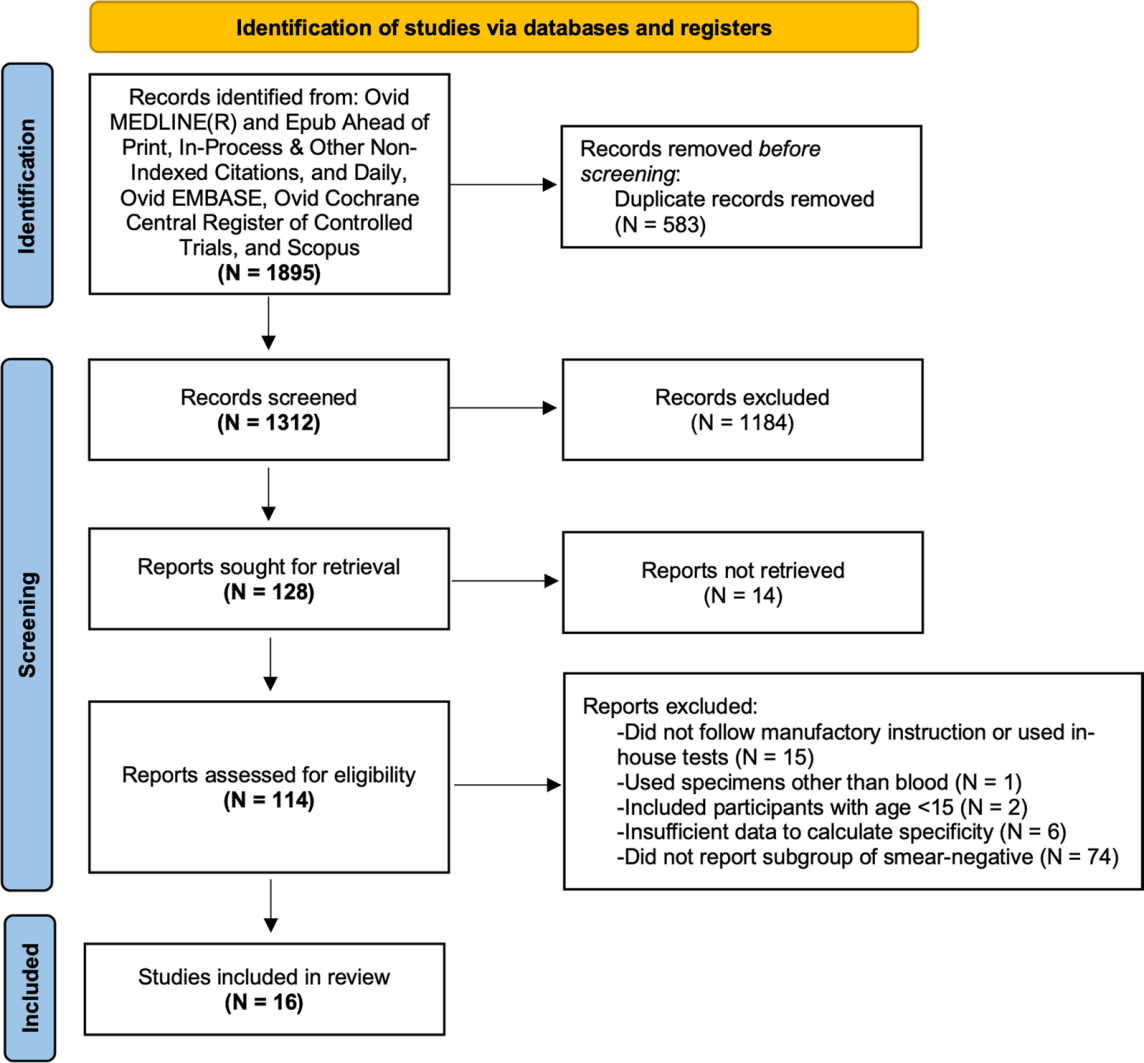
Study selection

Of 1,312 articles retrieved from the systematic search, 1,182 were excluded through title and abstract screening. Of 127 articles that underwent full-text review, a total of 16 studies were included in the final analysis (Fig. 1). The percent of agreement and $\kappa$ statistic for study selection were 95% and 0.77, respectively.

Fourteen studies [2–10, 23–27] reported the diagnostic accuracy of QFT-GIT, while 5 studies [10, 25, 26, 28, 29] reported the diagnostic accuracy of T-SPOT.TB. No study of QFT-GIT-plus was identified. A total of 1,204 and 2,658 SNPTB were included for QFT-GIT and T-SPOT.TB test, respectively. The studies were conducted in 10 different countries, of which 7 studies (44%) were conducted in high tuberculosis burden countries, according to the World Health Organization Global Tuberculosis Report 2020 [2, 3, 7, 9, 25, 28, 29]. Characteristics of included studies were demonstrated in Table 1. The diagnostic criteria for standard reference of active pulmonary tuberculosis in each study were described in Table 2. The absolute numbers of true positive, true negative, false positive, and false negative regarding SNPTB in each study are shown in Additional file : Table S2.

**Table 1:** Characteristic of included studies

Author	Year	Country	IGRAs	Study design	Smear negative/ total TB patients	Confirmed TB patients*N (%)	Immunocompromised		History of tuberculosis N (%)	History of BCG vaccination N (%)	Control subjects
							HIV N (%)	Non-HIV N (%)			
Cattamanchi et al	2010	Uganda	QFT	Prospective	39/126	126 (100)	236 (100)	0 (0)	NA	NA	Other diseases
Lee et al	2010	South Korea	QFT	Retrospective	32/32	32 (100)	NA	NA	18 (21)	NA	Other diseases
Leung et al	2010	Hongkong	QFT	Prospective	188/188	121 (64)	0 (0)	NA	18 (7)	NA	Other diseases
SA Kabeer et al	2010	India	QFT	Case control	26/177	162 (92)	0 (0)	0 (0)	0 (0)	NA	Healthy
Ling et al	2011	South Africa	Both	Prospective	46/138	138 (100)	108 (27)	NA	NA	NA	Other diseases
Lui et al	2011	Hongkong	QFT	Prospective	38/63	60 (95)	2 (1)	16 (9)	44(25)	88(70)	Other diseases
Jung et al	2012	South Korea	Both	Prospective	39/39	60 (95)	0 (0)	119 (100)	24 (20)	29 (30)	Other diseases
Taki-Eddin et al	2012	Syria	QFT	Unclear	13/48	40 (83)	NA	NA	NA	NA	Other diseases
Qian et al	2013	China	QFT	Case control	238/458	NA	NA	NA	NA	NA	Healthy
Park et al	2014	South Korea	QFT	Retrospective	94/94	69 (73)	0 (0)	13(14)	18(19.15)	NA	NA
Qiu et al	2015	China	T-SPOT	Unclear	382/517	224 (59)	0 (0)	NA	NA	NA	Other diseases and healthy
Xia et al	2015	China	QFT	Prospective	171/300	0 (0)	1 (0.3)	0 (0)	0 (0)	NA	Other diseases and healthy
Azghay et al	2016	France	QFT	Retrospective	23/60	39 (65)	5 (8)	5 (8)	NA	NA	Other diseases
Phetsuksiri et al	2018	Thailand	QFT	Case control	49/102	64 (63)	NA	NA	NA	NA	Healthy
Yang et al	2018	China	T-SPOT	Retrospective	2007/2282	640 (28)	0 (0)	86 (4)	NA	NA	Other diseases
Whitworth et al	2019	United Kingdom	Both	Prospective	222/363	261 (72)	135 (16)	17 (2)	NA	649 (77)	Other diseases

BCG, Bacillus Calmette-Guerin; HIV, human immunodeficiency virus; IGRAs, interferon-gamma release assays; QFT, QuantiFERON-TB Gold In-Tube; T-SPOT, T-SPOT.TB; TB, tuberculosis

*Confirmed TB patients defined by active pulmonary tuberculosis patients confirmed diagnosis with isolation of M. tuberculosis or histopathology of caseous granulomatous inflammation

**Table 2:** Diagnostic criteria for active pulmonary tuberculosis in each included study

Study	Isolation of *M. tuberculosis*	Caseous granulomatous inflammation in histopathology	Clinical improvement after anti-TB treatment
Cattamanchi et al	✓	X	X
Lee et al	✓	✓	✓
Leung et al	✓	X	✓
SA Kabeer et al	✓	X	✓
Ling et al	✓	X	X
Lui et al	✓	✓	✓
Jung et al	✓	X	✓
Taki-Eddin et al	✓	✓	✓
Qian et al	✓	✓	✓
Park et al	✓	✓	✓
Qiu et al	✓	X	✓
Xia et al	X	X	✓
Azghay et al	✓	✓	✓
Phetsuksiri et al	✓	X	✓
Yang et al	✓	✓	✓
Whitworth et al	✓	X	✓

TB, tuberculosis

### Study quality

**Fig. 2:**
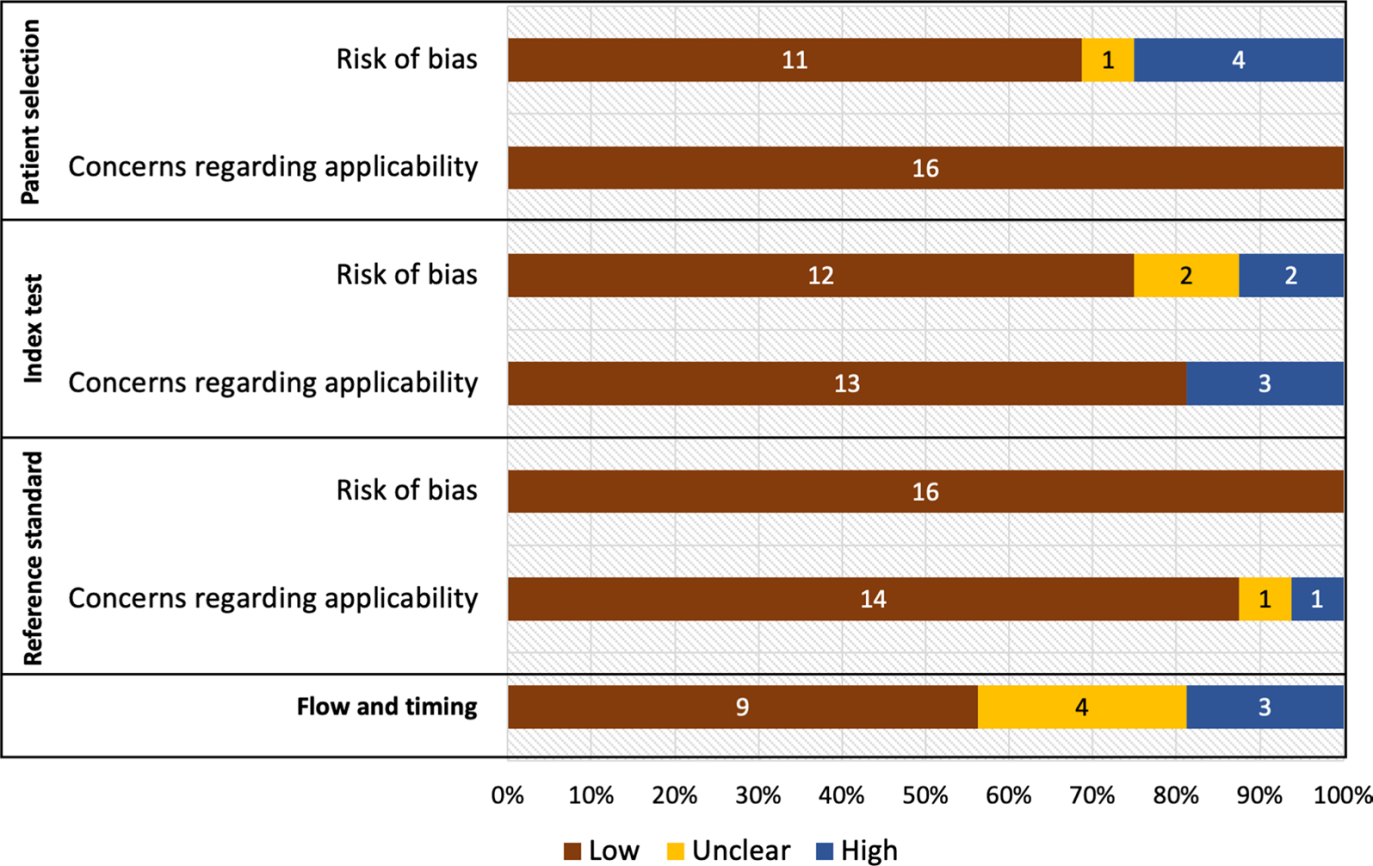
Summary of assessment of study quality using QUADAS-2 tool stratified by each QUADAS-2 item

Of 16 included studies, 8 (50%) studies meet the low risk of bias criteria. Of 8 studies having at risk of bias, 4 studies were conducted using case–control design. Other 3 studies had concerns regarding the applicability of IGRA test since they defined indeterminate results as negative IGRA test and 1 study defined patients with negative tuberculosis culture as non-tuberculosis group without mention of bronchoscopy even though the study was conducted in high tuberculosis burden area. The summary and details of the risk of bias assessment are demonstrated in Fig. 2 and Additional file 1: Table S3.

### Diagnostic accuracy

**Fig. 3:**
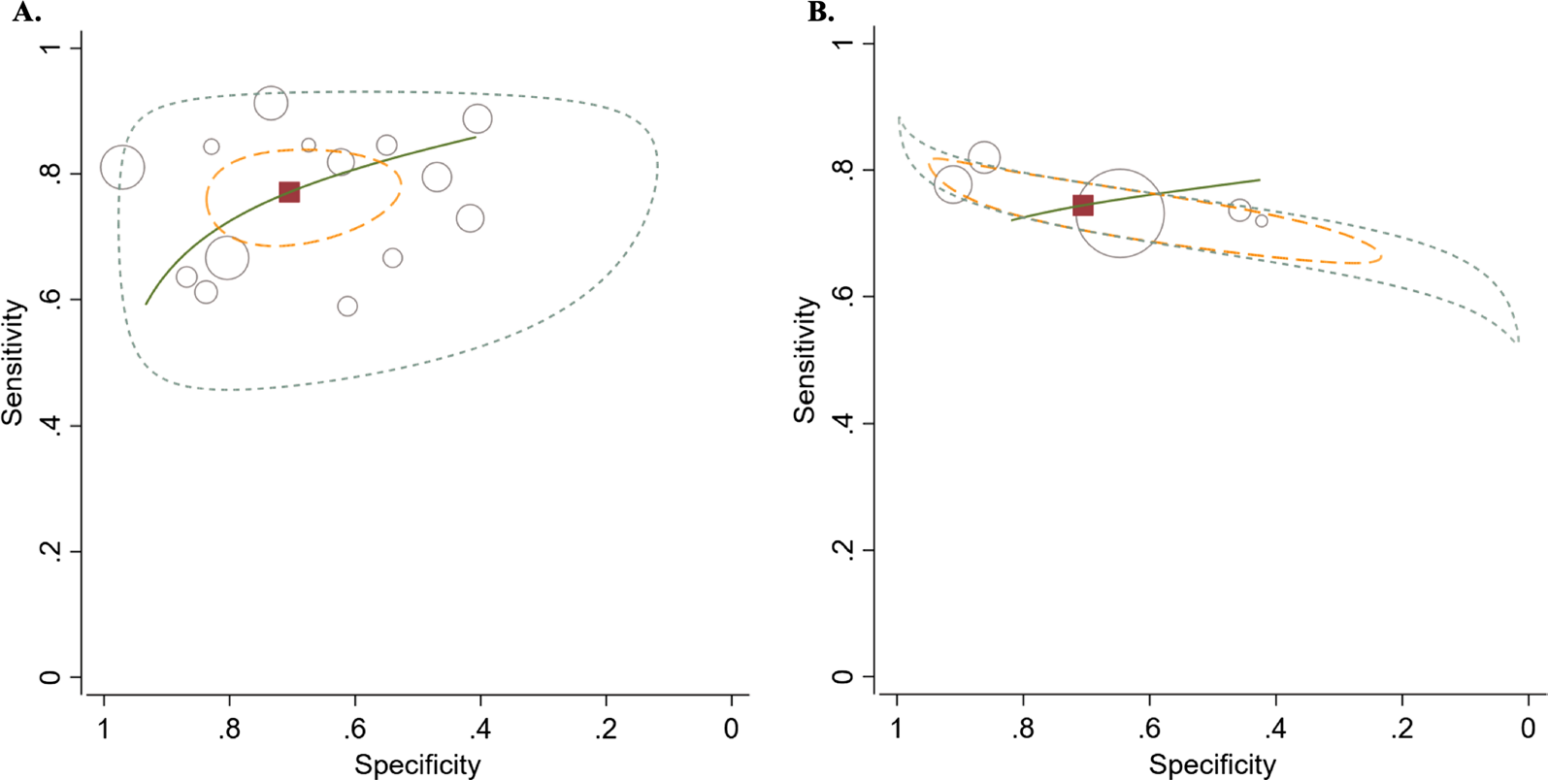
Hierarchical summary receiver operating characteristic (HSROC) plots demonstrate summary operating point (red square), 95% confidence interval (yellow dash line) and HSROC curve (green solid line) of **A** QuantiFERON-TB Gold In-Tube and **B** T-SPOT.TB for diagnosis of smear-negative pulmonary tuberculosis. Open circles represent individual study included in the meta-analysis, with circle size representing the sample size in each study

For diagnosis SNPTB, QFT-GIT had sensitivity of 0.77 (95% CI 0.71–0.82), specificity of 0.70 (95% CI 0.58–0.80), DOR of 8.03 (95% CI 4.51–14.31), positive LR of 2.61 (95% CI 1.80–3.80), negative LR of 0.33 (95% CI 0.25–0.42), and AUROC of 0.81 (95% CI 0.77–0.84) (Table 3 and Fig. 3). For T-SPOT.TB, the diagnostic accuracy included sensitivity of 0.74 (95% CI 0.71–0.78), specificity of 0.71 (95% CI 0.49–0.86), DOR of 6.96 (95% CI 2.31–20.98), positive LR of 2.53 (95% CI 1.26–5.07), negative LR of 0.36 (95% CI 0.24–0.55), and AUROC of 0.77 (95% CI 0.73–0.80) (Table 3 and Fig. 3).

**Table 3:** Diagnosis accuracy of QuantiFERON-TB Gold In-Tube and T-SPOT.TB

Test/Group	Sensitivity (95% CI)	Specificity (95% CI)	Positive LR (95% CI)	Negative LR (95% CI)	DOR (95% CI)
1. QFT-GIT					
1.1 Overall	0.77 (0.71–0.82)	0.70 (0.58–0.80)	2.61 (1.80–3.80)	0.33 (0.25–0.42)	8.03 (4.51–14.31)
1.2 Risk of bias					
Low risk of bias	0.77 (0.69–0.83)	0.69 (0.56–0.79)	2.46 (1.83–3.31)	0.34 (0.28–0.41)	7.28 (5.39–9.85)
At risk of bias	0.76 (0.68–0.82)	0.71 (0.50–0.86)	2.64 (1.36–5.12)	0.34 (0.22–0.51)	7.85 (2.81–21.90)
1.3 TB burden					
High TB burden country	0.74 (0.66–0.81)	0.57 (0.42–0.71)	1.74 (1.30–2.33)	0.45 (0.36–56)	3.84 (2.45–6.02)
Low TB burden country	0.80 (0.71–0.85)	0.77 (0.62–0.87)	3.39 (2.02–5.68)	0.27 (0.19–0.39)	12.38 (5.92–25.89)
1.4 Excluding studies of patients with HIV and immunocompromised status	0.79 (0.73–0.84)	0.72 (0.59–0.83)	2.86 (1.86–4.40)	0.29 (0.22–0.38)	8.85 (5.36–18.11)
2.T-SPOT.TB	0.74 (0.71–0.78)	0.71 (0.49–0.86)	2.53 (1.26–5.07)	0.36 (0.24–0.55)	6.96 (2.31–20.98)

CI, confidence interval; DOR, diagnostic odds ratio; HIV, human immunodeficiency virus; LR likelihood ratio; QFT-GIT, QuantiFERON-TB Gold In-Tube; TB, tuberculosis

### Subgroup and sensitivity analyses

The sensitivity and negative LR of QFT-GIT appeared consistence in almost subgroup analyses. However, the specificity, positive LR, and DOR seemed lower in the subgroup of studies conducted in high tuberculosis burden countries compared to the low tuberculosis burden countries. The sensitivity analysis by excluding studies conducted in patients with HIV infection and immunocompromised status demonstrated the robustness of diagnostic accuracy of QFT-GIT for smear-negative pulmonary tuberculosis, with sensitivity of 0.79 (95% CI 0.73–0.84), specificity of 0.72 (95% CI 0.59–0.83), DOR of 8.85 (95% CI 5.36–18.11), positive LR of 2.86 (95% CI 1.86–4.40), negative LR of 0.29 (95% CI 0.22–0.38) (Table 3).

## Discussion

Our systematic review and meta-analysis revealed comparable diagnostic performance for SNPTB diagnosis between QFT-GIT and T-SPOT.TB. However, both tests appear insufficient for ruling in or ruling out SNPTB. The sensitivities and specificities for QFT-GIT and T-SPOT.TB were in the 0.7–0.8 ranges. The specificity of QFT-GIT was lower in the subgroup of studies conducted in high tuberculosis burden areas, with the specificity of 0.57 (95% CI; 0.42–0.71).

Previous studies also demonstrated the moderate diagnostic performance of IGRAs for active pulmonary tuberculosis, including positive-smear patients [14, 15]. Our study revealed similar diagnostic performance in SNPTB despite potentially having a lower mycobacterial burden. Our findings emphasize that IGRAs may not correlate with sputum smear. The previous study using in-house IGRAs revealed that the positive rate of IGRAs was not significantly different between patients with positive and negative sputum smears [30]. In contrast to our study, previous systematic reviews and meta-analyses demonstrated T-SPOT.TB had lower specificity than QFT-GIT for active tuberculosis diagnosis [14]. The difference in inclusion criteria might explain the contradiction as the former meta-analysis included studies of LTBI. The involvement of LTBI may not represent the diagnosis algorithm in routine practice and result in an underestimation of specificity.

The diagnostic performance of IGRAs for diagnosis of SNPTB seems not to be high enough to diagnose or rule out active tuberculosis infection. As the IFN-γ can be detected in any stage of broad-spectrum tuberculosis diseases, the tests cannot distinguish active versus latent infection, particularly in areas of a high prevalence of tuberculosis [31]. Previous systematic reviews and meta-analyses demonstrated lower specificity of IGRAs for diagnosis of active tuberculosis in countries with low and middle income [13, 31]. Our study showed the robustness of the result since the specificity of QFT-GIT was lower in the subgroup analysis of high tuberculosis burden countries. The IGRAs may not be beneficial in this situation.

Our study reported the pooled sensitivity of 0.77 and 0.74 for QFT-GIT and T-SPOT. TB, respectively. That means approximately 25% of patients with SNPTB had negative IGRA results. As described above, IGRAs measure levels of IFN-γ released from T-cell lymphocytes. Several factors affecting T-cell function might be associated with false-negative IGRA results. Advanced age and low peripheral lymphocyte counts have been proposed as risk factors associated with false-negative IGRA results [22]. Low peripheral lymphocyte counts are reasonable to associate with a decrease in the production of IFN-γ response to specific Mycobacterium antigens. Although low peripheral lymphocyte counts may correlate to advanced age, previous studies demonstrated that advanced age was associated with false-negative T-SPOT.TB under the optimization of lymphocyte counts [32]. HIV infection was also associated with false-negative IGRAs [22]. As observed in our study, the overall sensitivity was higher than previous studies conducted on the HIV population [2]. The mechanism of the association remains controversial as it is unclear whether the CD4^+^ count directly affects the performance of IGRAs [33].

The main limitation we encountered conducting this review is the heterogeneity of the diagnostic gold standard for SNPTB. We cannot emphasize enough the diagnostic challenge of SNPTB. In our clinical practice, patients with negative acid-fast stain should undergo further diagnostic procedures, such as bronchoscopy, to obtain adequate specimens for mycobacterial culture. Unfortunately, the procedure might not be practical in some clinical settings. Therefore, our study included a variety of gold standard definitions. This may result in over-or under-estimation of the diagnostic accuracy.

There are other limitations worth noting. First, the study of QFT-plus was not included. QFT-plus might have higher diagnostic accuracy than QFT-GIT and T-SPOT.TB. One meta-analysis showed that the sensitivity of the QFT-plus for detecting tuberculosis infection is higher than QFT-GIT [34]. The higher sensitivity of QFT-plus because this test can detect both CD4 and CD8 T-cell responses. Further studies focusing on evaluating the accuracy of QFT-plus on smear-negative pulmonary tuberculosis is warranted. Second, only 50% of included studies meet the low risk of bias criteria. Nevertheless, subgroup analysis showed no difference in results between the low-risk and at-risk biases groups. Finally, because of the low number of T-SPOT.TB in included studies, so we cannot do subgroup analysis regarding the risk of bias, tuberculosis burden, and proportion of confirmed cases.

## Conclusions

Our systematic review and meta-analysis showed that IGRAs have suboptimal accuracy for diagnosing or ruling out SNPTB. Nevertheless, IGRAs might be valuable tests for excluding tuberculosis among patients with low pre-test probability, especially in countries with a low tuberculosis burden.

## Supplementary Information

**Additional file 1:** **TableS1.** Actual search strategies. **TableS2.** Absolutenumber of true positive, true negative, false positive, and falsenegative in each study. **TableS3.** Riskof bias assessment for included studies.

## Data Availability

The datasets used and/or analysed during the current study available from the corresponding author on reasonable request.

## Abbreviations

AUROC: Area under receiver operating characteristic
DOR: Diagnostic odds ratio
HSROC: Hierarchical summary receiver operating characteristic
IFN-γ: Interferon-gamma
IGRAs: Interferon gamma-release assays
LR: Likelihood ratio
LTBI: Latent tuberculosis infection
QFT-GIT: QuantiFERON-TB Gold In-Tube
QFT-plus: QuantiFERON-TB Gold Plus
SNPTB: Smear-negative pulmonary tuberculosis

## References

[1] Global tuberculosis report 2021. https://www.who.int/teams/global-tuberculosis-programme/tb-reports/global-tuberculosis-report-2021.

[2] Cattamanchi A, Ssewenyana I, Davis JL, Huang L, Worodria W, den Boon S, Yoo S, Andama A, Hopewell PC, Cao H (2010). Role of interferon-gamma release assays in the diagnosis of pulmonary tuberculosis in patients with advanced HIV infection. BMC Infect Dis.

[3] Syed Ahamed Kabeer B, Raman B, Thomas A, Perumal V, Raja A (2010). Role of QuantiFERON-TB gold, interferon gamma inducible protein-10 and tuberculin skin test in active tuberculosis diagnosis. PLoS ONE.

[4] Lui G, Lee N, Cheung SW, Lam JSY, Wong BCK, Choi KW, Wong KT, Wong RYK, Cockram CS, Hui DSC (2011). Interferon gamma release assay for differentiating tuberculosis among pneumonia cases in acute healthcare setting. J Infect.

[5] Taki-Eddin L, Monem F (2012). Utility of an interferon-gamma release assay as a potential diagnostic aid for active pulmonary tuberculosis. J Infect Dev Ctries.

[6] Qian F, Wang W, Qiu Z, Shen Y, He J, Li D, Zhong J, Dai L (2013). Evaluation of a new tuberculosis-related interferon gamma release assay for tuberculosis infection diagnosis in Huzhou, eastern China. Indian J Pathol Microbiol.

[7] Xia H, Wang X, Li F, Longuet C, Vernet G, Goletti D, Zhao Y, Lagrange PH (2015). Diagnostic values of the QuantiFERON-TB Gold In-tube assay carried out in China for diagnosing pulmonary tuberculosis. PLoS ONE.

[8] Azghay M, Bouchaud O, Mechai F, Nicaise P, Fain O, Stirnemann J (2016). Utility of QuantiFERON-TB Gold In-Tube assay in adult, pulmonary and extrapulmonary, active tuberculosis diagnosis. Int J Infect Dis.

[9] Phetsuksiri B, Srisungngam S, Rudeeaneksin J, Boonchu S, Klayut W, Norrarat R, Sangkitporn S, Kasetjaroen Y (2018). QuantiFERON-TB Gold In-Tube test in active tuberculosis patients and healthy adults. Rev Inst Med Trop Sao Paulo.

[10] Whitworth HS, Badhan A, Boakye AA, Takwoingi Y, Rees-Roberts M, Partlett C, Lambie H, Innes J, Cooke G, Lipman M (2019). Clinical utility of existing and second-generation interferon-gamma release assays for diagnostic evaluation of tuberculosis: an observational cohort study. Lancet Infect Dis.

[11] Pai M, Denkinger CM, Kik SV, Rangaka MX, Zwerling A, Oxlade O, Metcalfe JZ, Cattamanchi A, Dowdy DW, Dheda K (2014). Gamma interferon release assays for detection of Mycobacterium tuberculosis infection. Clin Microbiol Rev.

[12] Diel R, Goletti D, Ferrara G, Bothamley G, Cirillo D, Kampmann B, Lange C, Losi M, Markova R, Migliori GB (2011). Interferon-gamma release assays for the diagnosis of latent Mycobacterium tuberculosis infection: a systematic review and meta-analysis. Eur Respir J.

[13] Metcalfe JZ, Everett CK, Steingart KR, Cattamanchi A, Huang L, Hopewell PC, Pai M (2011). Interferon-gamma release assays for active pulmonary tuberculosis diagnosis in adults in low- and middle-income countries: systematic review and meta-analysis. J Infect Dis.

[14] Sester M, Sotgiu G, Lange C, Giehl C, Girardi E, Migliori GB, Bossink A, Dheda K, Diel R, Dominguez J (2011). Interferon-gamma release assays for the diagnosis of active tuberculosis: a systematic review and meta-analysis. Eur Respir J.

[15] Lu P, Chen X, Zhu LM, Yang HT (2016). Interferon-gamma release assays for the diagnosis of tuberculosis: a systematic review and meta-analysis. Lung.

[16] Ma Y, Xu Y, Cao X, Chen X, Zhong Y (2021). Diagnostic value of interferon-gamma release assay in HIV-infected individuals complicated with active tuberculosis: a systematic review and meta-analysis. Epidemiol Infect.

[17] Fan L, Chen Z, Hao XH, Hu ZY, Xiao HP (2012). Interferon-gamma release assays for the diagnosis of extrapulmonary tuberculosis: a systematic review and meta-analysis. FEMS Immunol Med Microbiol.

[18] Aggarwal AN, Agarwal R, Gupta D, Dhooria S, Behera D (2015). Interferon gamma release assays for diagnosis of pleural tuberculosis: a systematic review and meta-analysis. J Clin Microbiol.

[19] Whiting PF, Rutjes AW, Westwood ME, Mallett S, Deeks JJ, Reitsma JB, Leeflang MM, Sterne JA, Bossuyt PM (2011). Group Q-: QUADAS-2: a revised tool for the quality assessment of diagnostic accuracy studies. Ann Intern Med.

[20] Rutter CM, Gatsonis CA (2001). A hierarchical regression approach to meta-analysis of diagnostic test accuracy evaluations. Stat Med.

[21] Nguyen DT, Teeter LD, Graves J, Graviss EA (2018). Characteristics associated with negative interferon-gamma release assay results in culture-confirmed tuberculosis patients, Texas, USA, 2013–2015. Emerg Infect Dis.

[22] Yamasue M, Komiya K, Usagawa Y, Umeki K, Nureki SI, Ando M, Hiramatsu K, Nagai H, Kadota JI (2020). Factors associated with false negative interferon-gamma release assay results in patients with tuberculosis: A systematic review with meta-analysis. Sci Rep.

[23] Lee H-M, Shin JW, Kim JY, Park IW, Choi BW, Choi JC, Seo JS, Kim CW (2010). HRCT and whole-blood interferon-gamma assay for the rapid diagnosis of smear-negative pulmonary tuberculosis. Respiration.

[24] Leung ECC, Leung CC, Leung WWL, Kam KM, Yew WW, Lee SN, Tam CM (2010). Role of whole-blood interferon-gamma release assay in the diagnosis of smear-negative tuberculosis. Int J Tuberc Lung Dis.

[25] Ling DI, Pai M, Davids V, Brunet L, Lenders L, Meldau R, Calligaro G, Allwood B, van Zyl-Smit R, Peter J (2011). Are interferon-gamma release assays useful for diagnosing active tuberculosis in a high-burden setting?. Eur Respir J.

[26] Jung JY, Lim JE, Lee H-J, Kim YM, Cho S-N, Kim SK, Chang J, Kang YA (2012). Questionable role of interferon-gamma assays for smear-negative pulmonary TB in immunocompromised patients. J Infect.

[27] Park H, Shin JA, Kim HJ, Ahn CM, Chang YS (2014). Whole blood interferon-gamma release assay is insufficient for the diagnosis of sputum smear negative pulmonary tuberculosis. Yonsei Med J.

[28] Qiu Y, Wang Y, Lin N, Huang M, Tan Y, Wang Q, Jiang Y, Liu H, Liu J, Zhang J (2015). Multicenter clinical evaluation of three commercial reagent kits based on the interferon-gamma release assay for the rapid diagnosis of tuberculosis in China. Int J Infect Dis.

[29] Yang C, Zhang S, Yao L, Fan L (2018). Evaluation of risk factors for false-negative results with an antigen-specific peripheral blood-based quantitative T cell assay (T-SPOT.TB) in the diagnosis of active tuberculosis: a large-scale retrospective study in China. J Int Med Res.

[30] Ji L, Lou YL, Wu ZX, Jiang JQ, Fan XL, Wang LF, Liu XX, Du P, Yan J, Sun AH (2017). Usefulness of interferon-gamma release assay for the diagnosis of sputum smear-negative pulmonary and extra-pulmonary TB in Zhejiang Province, China. Infect Dis Poverty.

[31] Aabye MG, Latorre I, Diaz J, Maldonado J, Mialdea I, Eugen-Olsen J, Ravn P, Dominguez J, Ruhwald M (2013). Dried plasma spots in the diagnosis of tuberculosis: IP-10 release assay on filter paper. Eur Respir J.

[32] Lian G, Du F, Wu H, He M, Liu Z (2017). Factors contributing to false-negative enzyme-linked immunospot assay for interferon-gamma results in active tuberculosis. Clin Lab.

[33] Santin M, Munoz L, Rigau D (2012). Interferon-gamma release assays for the diagnosis of tuberculosis and tuberculosis infection in HIV-infected adults: a systematic review and meta-analysis. PLoS ONE.

[34] Sotgiu G, Saderi L, Petruccioli E, Aliberti S, Piana A, Petrone L, Goletti D (2019). QuantiFERON TB Gold Plus for the diagnosis of tuberculosis: a systematic review and meta-analysis. J Infect.

