# Supplementary material for: Diagnostic accuracy of interferon-gamma release assays for diagnosis of smear-negative pulmonary tuberculosis: a systematic review and meta-analysis
Source: BMC Pulm Med. 2022 Jun 6;22:219. doi: 10.1186/s12890-022-02013-y (PMC9169405; doi:10.1186/s12890-022-02013-y)
Supplement: Supplementary file 1 — Additional file 1: TableS1. Actual search strategies. TableS2. Absolutenumber of true positive, true negative, false positive, and falsenegative in each study. TableS3. Riskof bias assessment for included studies. [file 12890_2022_2013_MOESM1_ESM.docx]

**Table S1: Actual search strategies**

**OVID**

Database(s): EBM Reviews - Cochrane Central Register of Controlled Trials February 2021, EBM Reviews - Cochrane Database of Systematic Reviews 2005 to March 31, 2021, Embase 1974 to 2021 April 02 , Ovid MEDLINE(R) and Epub Ahead of Print, In-Process, In-Data-Review & Other Non-Indexed Citations and Daily 1946 to April 02, 2021

| **#** | **Searches** | **Results** |
| --- | --- | --- |
| 1 | exp Tuberculosis/ | 395812 |
| 2 | ("koch disease" or "kochs disease" or TB or tuberculoses or tuberculosis).ti,ab,hw,kw. | 550697 |
| 3 | 1 or 2 | 562172 |
| 4 | (active or "Consecutive smear*" or "E-TB" or "extrapulmonary tuberculosis" or "extra-pulmonary tuberculosis" or "RIF-positive" or "Xpert R MTB").ti,ab,hw,kw. | 2509837 |
| 5 | 3 and 4 | 58213 |
| 6 | exp Interferon-gamma Release Tests/ | 5412 |
| 7 | (ELISPOT or "enzyme-linked immunospot assay" or "enzyme-linked immunospot assays" or "GFT-GIT" or IGRA or IGRAs or "interferon-gamma release assay" or "interferon-gamma release assays" or "interferon-gamma release test" or "Interferon-gamma Release Tests" or "QFT-GIT" or "QFT-IT" or "QFT-Plus" or "QuantiFERONR-TB" or "QuantiFERONR-TB Gold" or "QuantiFERONR-TB Gold In-Tube" or "QuantiFERONR-TB Gold Plus" or "QuantiFERON-TB" or "QuantiFERON-TB Gold" or "QuantiFERON-TB Gold In-Tube" or "QuantiFERON-TB Gold Plus" or "T-SPOT TB" or "T-SPOT*").ti,ab,hw,kw. | 33311 |
| 8 | 6 or 7 | 33311 |
| 9 | 5 and 8 | 4345 |
| 10 | exp "Sensitivity and Specificity"/ | 1009083 |
| 11 | ((diagnos* adj3 (accuracy or usefulness)) or predict* or sensitivit* or specificit*).ti,ab,hw,kw. | 7682301 |
| 12 | 10 or 11 | 7722062 |
| 13 | 9 and 12 | 2175 |
| 14 | (exp animals/ or exp nonhuman/) not exp humans/ | 11406725 |
| 15 | ((alpaca or alpacas or amphibian or amphibians or animal or animals or antelope or armadillo or armadillos or avian or baboon or baboons or beagle or beagles or bee or bees or bird or birds or bison or bovine or buffalo or buffaloes or buffalos or "c elegans" or "Caenorhabditis elegans" or camel or camels or canine or canines or carp or cats or cattle or chick or chicken or chickens or chicks or chimp or chimpanze or chimpanzees or chimps or cow or cows or "D melanogaster" or "dairy calf" or "dairy calves" or deer or dog or dogs or donkey or donkeys or drosophila or "Drosophila melanogaster" or duck or duckling or ducklings or ducks or equid or equids or equine or equines or feline or felines or ferret or ferrets or finch or finches or fish or flatworm or flatworms or fox or foxes or frog or frogs or "fruit flies" or "fruit fly" or "G mellonella" or "Galleria mellonella" or geese or gerbil or gerbils or goat or goats or goose or gorilla or gorillas or hamster or hamsters or hare or hares or heifer or heifers or horse or horses or insect or insects or jellyfish or kangaroo or kangaroos or kitten or kittens or lagomorph or lagomorphs or lamb or lambs or llama or llamas or macaque or macaques or macaw or macaws or marmoset or marmosets or mice or minipig or minipigs or mink or minks or monkey or monkeys or mouse or mule or mules or nematode or nematodes or octopus or octopuses or orangutan or "orang-utan" or orangutans or "orang-utans" or oxen or parrot or parrots or pig or pigeon or pigeons or piglet or piglets or pigs or porcine or primate or primates or quail or rabbit or rabbits or rat or rats or reptile or reptiles or rodent or rodents or ruminant or ruminants or salmon or sheep or shrimp or slug or slugs or swine or tamarin or tamarins or toad or toads or trout or urchin or urchins or vole or voles or waxworm or waxworms or worm or worms or xenopus or "zebra fish" or zebrafish) not (human or humans or patient or patients)).ti,ab,hw,kw. | 9810895 |
| 16 | exp Models, Animal/ | 2072669 |
| 17 | 13 not (14 or 15 or 16) | 2159 |
| 18 | limit 17 to ("all adult (19 plus years)" or "young adult (19 to 24 years)" or "adult (19 to 44 years)" or "young adult and adult (19-24 and 19-44)" or "middle age (45 to 64 years)" or "middle aged (45 plus years)" or "all aged (65 and over)" or "aged (80 and over)") [Limit not valid in CCTR,CDSR,Embase; records were retained] | 1831 |
| 19 | limit 18 to (adult <18 to 64 years> or aged <65+ years>) [Limit not valid in CCTR,CDSR,Ovid MEDLINE(R),Ovid MEDLINE(R) Daily Update,Ovid MEDLINE(R) PubMed not MEDLINE,Ovid MEDLINE(R) In-Process,Ovid MEDLINE(R) Publisher; records were retained] | 1168 |
| 20 | limit 17 to ("all infant (birth to 23 months)" or "all child (0 to 18 years)" or "newborn infant (birth to 1 month)" or "infant (1 to 23 months)" or "preschool child (2 to 5 years)" or "child (6 to 12 years)" or "adolescent (13 to 18 years)") [Limit not valid in CCTR,CDSR,Embase; records were retained] | 1653 |
| 21 | limit 20 to (embryo or infant or child or preschool child <1 to 6 years> or school child <7 to 12 years> or adolescent <13 to 17 years>) [Limit not valid in CCTR,CDSR,Ovid MEDLINE(R),Ovid MEDLINE(R) Daily Update,Ovid MEDLINE(R) PubMed not MEDLINE,Ovid MEDLINE(R) In-Process,Ovid MEDLINE(R) Publisher; records were retained] | 463 |
| 22 | 21 not 19 | 192 |
| 23 | 17 not 22 | 1967 |
| 24 | (case adj3 report).mp,pt. | 3198553 |
| 25 | 23 not 24 | 1925 |
| 26 | limit 25 to (editorial or erratum or note or addresses or autobiography or bibliography or biography or blogs or comment or dictionary or directory or interactive tutorial or interview or lectures or legal cases or legislation or news or newspaper article or overall or patient education handout or periodical index or portraits or published erratum or video-audio media or webcasts) [Limit not valid in CCTR,CDSR,Embase,Ovid MEDLINE(R),Ovid MEDLINE(R) Daily Update,Ovid MEDLINE(R) PubMed not MEDLINE,Ovid MEDLINE(R) In-Process,Ovid MEDLINE(R) Publisher; records were retained] | 30 |
| 27 | 25 not 26 | 1895 |
| 28 | remove duplicates from 27 | 1326 |

**Scopus**

| 1 | TITLE-ABS-KEY("koch disease" OR "kochs disease" OR TB OR tuberculoses OR tuberculosis) |
| --- | --- |
| 2 | TITLE-ABS-KEY(active OR "Consecutive smear*" OR "E-TB" OR "extrapulmonary tuberculosis" OR "extra-pulmonary tuberculosis" OR "RIF-positive" OR "Xpert R MTB") |
| 3 | TITLE-ABS-KEY(ELISPOT OR "enzyme-linked immunospot assay" OR "enzyme-linked immunospot assays" OR "GFT-GIT" OR IGRA OR IGRAs OR "interferon-gamma release assay" OR "interferon-gamma release assays" OR "interferon-gamma release test" OR "Interferon-gamma Release Tests" OR "QFT-GIT" OR "QFT-IT" OR "QFT-Plus" OR "QuantiFERONR-TB" OR "QuantiFERONR-TB Gold" OR "QuantiFERONR-TB Gold In-Tube" OR "QuantiFERONR-TB Gold Plus" OR "QuantiFERON-TB" OR "QuantiFERON-TB Gold" OR "QuantiFERON-TB Gold In-Tube" OR "QuantiFERON-TB Gold Plus" OR "T-SPOT TB" OR "T-SPOT*") |
| 4 | TITLE-ABS-KEY((diagnos* W/3 (accuracy or usefulness)) OR predict* OR sensitivit* OR specificit*) |
| 5 | 1 and 2 and 3 and 4 |
| 6 | TITLE-ABS-KEY((alpaca OR alpacas OR amphibian OR amphibians OR animal OR animals OR antelope OR armadillo OR armadillos OR avian OR baboon OR baboons OR beagle OR beagles OR bee OR bees OR bird OR birds OR bison OR bovine OR buffalo OR buffaloes OR buffalos OR "c elegans" OR "Caenorhabditis elegans" OR camel OR camels OR canine OR canines OR carp OR cats OR cattle OR chick OR chicken OR chickens OR chicks OR chimp OR chimpanze OR chimpanzees OR chimps OR cow OR cows OR "D melanogaster" OR "dairy calf" OR "dairy calves" OR deer OR dog OR dogs OR donkey OR donkeys OR drosophila OR "Drosophila melanogaster" OR duck OR duckling OR ducklings OR ducks OR equid OR equids OR equine OR equines OR feline OR felines OR ferret OR ferrets OR finch OR finches OR fish OR flatworm OR flatworms OR fox OR foxes OR frog OR frogs OR "fruit flies" OR "fruit fly" OR "G mellonella" OR "Galleria mellonella" OR geese OR gerbil OR gerbils OR goat OR goats OR goose OR gorilla OR gorillas OR hamster OR hamsters OR hare OR hares OR heifer OR heifers OR horse OR horses OR insect OR insects OR jellyfish OR kangaroo OR kangaroos OR kitten OR kittens OR lagomorph OR lagomorphs OR lamb OR lambs OR llama OR llamas OR macaque OR macaques OR macaw OR macaws OR marmoset OR marmosets OR mice OR minipig OR minipigs OR mink OR minks OR monkey OR monkeys OR mouse OR mule OR mules OR nematode OR nematodes OR octopus OR octopuses OR orangutan OR "orang-utan" OR orangutans OR "orang-utans" OR oxen OR parrot OR parrots OR pig OR pigeon OR pigeons OR piglet OR piglets OR pigs OR porcine OR primate OR primates OR quail OR rabbit OR rabbits OR rat OR rats OR reptile OR reptiles OR rodent OR rodents OR ruminant OR ruminants OR salmon OR sheep OR shrimp OR slug OR slugs OR swine OR tamarin OR tamarins OR toad OR toads OR trout OR urchin OR urchins OR vole OR voles OR waxworm OR waxworms OR worm OR worms OR xenopus OR "zebra fish" OR zebrafish) AND NOT (human OR humans or patient or patients)) |
| 7 | 5 and not 6 |
| 8 | TITLE-ABS-KEY(newborn* or neonat* or infant* or toddler* or child* or adolescent* or paediatric* or pediatric* or girl or girls or boy or boys or teen or teens or teenager* or preschooler* or "pre-schooler*" or preteen or preteens or "pre-teen" or "pre-teens" or youth or youths) AND NOT TITLE-ABS-KEY(adult or adults or "middle age" or "middle aged" OR elderly OR geriatric* OR "old people" OR "old person*" OR "older people" OR "older person*" OR "very old") |
| 9 | 7 and not 8 |
| 10 | TITLE-ABS-KEY(case W/3 report) |
| 11 | 9 and not 10 |
| 12 | DOCTYPE(ab) OR DOCTYPE(ed) OR DOCTYPE(bk) OR DOCTYPE(er) OR DOCTYPE(no) OR DOCTYPE(sh) |
| 13 | 11 and not 12 |
| 14 | INDEX(embase) OR INDEX(medline) OR PMID(0* OR 1* OR 2* OR 3* OR 4* OR 5* OR 6* OR 7* OR 8* OR 9*) |
| 15 | 13 and not 14 |

**Table S2:** Absolute number of true positive, true negative, false positive, and false negative in each study

2.1 QuantiFERON Gold-In-Tube

| **Study** | **True positive** | **True negative** | **False positive** | **False negative** |
| --- | --- | --- | --- | --- |
| Cattamanchi et al. | 26 | 40 | 34 | 13 |
| Lee et al. | 27 | 34 | 7 | 5 |
| Leung et al. | 167 | 30 | 44 | 21 |
| SA Kabeer et al. | 22 | 55 | 45 | 4 |
| Ling et al. | 27 | 83 | 116 | 10 |
| Lui et al. | 21 | 86 | 13 | 12 |
| Jung et al. | 23 | 49 | 31 | 16 |
| Taki-Eddin et al. | 11 | 29 | 14 | 2 |
| Qian et al. | 193 | 367 | 11 | 45 |
| Park et al. | 77 | 81 | 49 | 17 |
| Xia et al. | 136 | 47 | 53 | 35 |
| Azghay et al. | 21 | 246 | 89 | 2 |
| Phetsuksiri et al. | 30 | 93 | 18 | 19 |
| Whitworth et al. | 148 | 304 | 74 | 74 |

2.2 T-SPOT.TB

| **Study** | **True positive** | **True negative** | **False positive** | **False negative** |
| --- | --- | --- | --- | --- |
| Ling et al. | 28 | 108 | 128 | 10 |
| Jung et al. | 18 | 22 | 30 | 7 |
| Qiu et al. | 297 | 391 | 38 | 85 |
| Yang et al. | 1469 | 1643 | 896 | 538 |
| Whitworth et al. | 169 | 319 | 51 | 37 |

**Table S3:** Risk of bias assessment for included studies

| **Study** | **Patient selection** | | **Index test** | | **Reference standard** | | **Flow and timing** | **Overall** |
| --- | --- | --- | --- | --- | --- | --- | --- | --- |
|  | **ROB** | **Applicability** | **ROB** | **Applicability** | **ROB** | **Applicability** |  |  |
| Cattamanchi et al. | low | low | low | low | low | low | low | **low** |
| Lee et al. | low | low | low | low | low | low | low | **low** |
| Leung et al. | low | low | low | low | low | low | low | **low** |
| SA Kabeer et al. | high | low | low | low | low | low | unclear | **at risk** |
| Ling et al. | low | low | low | low | high | low | low | **at risk** |
| Lui et al. | low | low | low | low | low | low | low | **low** |
| Jung et al. | low | low | low | high | low | low | high | **at risk** |
| Taki-Eddin et al. | low | low | low | low | low | low | low | **low** |
| Qian et al. | high | low | low | low | unclear | unclear | low | **at risk** |
| Park et al. | low | low | low | low | low | low | low | **low** |
| Qui et al. | high | low | low | low | low | low | unclear | **at risk** |
| Xia et al. | unclear | low | low | high | high | high | low | **at risk** |
| Azghay et al. | low | low | low | high | low | low | unclear | **at risk** |
| Phetsuksiri et al. | high | low | low | low | low | low | low | **at risk** |
| Yang et al. | low | low | low | low | low | low | low | **low** |
| Whitworth et al. | low | low | low | low | low | low | low | **low** |
